# 1-{(*Z*)-[3-(1-Hy­droxy­eth­yl)anilino]methyl­idene}naphthalen-2(1*H*)-one

**DOI:** 10.1107/S1600536812050635

**Published:** 2012-12-19

**Authors:** Peter N. Horton, Mehmet Akkurt, Shaaban K. Mohamed, Antar A. Abdelhamid, Adel A. Marzouk

**Affiliations:** a School of Chemistry, University of Southampton, Highfield, Southampton, SO17 1BJ England, England; b Department of Physics, Faculty of Sciences, Erciyes University, 38039 Kayseri, Turkey; c Chemistry and Environmental Division, Manchester Metropolitan University, Manchester M1 5GD, England; d Chemistry Department, Faculty of Science, Minia University, El-Minia, Egypt; e Pharmaceutical Chemistry Department, Faculty of Pharmacy, Al Azhar University, Egypt

## Abstract

In the title compound, C_19_H_17_NO_2_, the dihedral angle between the benzene ring and the naphthalene ring system is 9.72 (5)°, while the torsion angle of the C—N—C—C bridging group is 179.24 (17)°. The methyl group of the 1-phenyl­ethanol moiety is disordered over two positions with a refined occupancy ratio of 0.775 (5):0.225 (5). The mol­ecular conformation is stabil­ized by an intra­molecular N—H⋯O hydrogen bond, which generates an *S*(6) ring motif. In the crystal, mol­ecules are linked by O—H⋯O hydrogen bonds, forming zigzag chains propagating along the *c-*axis direction. Neighbouring chains are linked *via* C—H⋯O inter­actions, forming a two-dimensional slab-like network parallel to the *bc* plane.

## Related literature

For the biological and industrial properties of Schiff bases, see: Keypour *et al.* (2009 ▶); Suslick & Reinert (1988 ▶); Tisato *et al.* (1994 ▶). For the synthesis and coordination chemistry of azomethines, see, for example: Singh & Adhikari (2012 ▶). For standard bond lengths, see: Allen *et al.* (1987 ▶). For hydrogen-bond motifs, see: Bernstein *et al.* (1995 ▶).
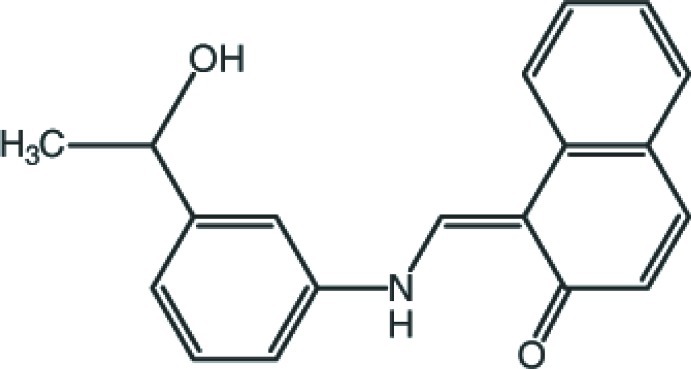


## Experimental

### 

#### Crystal data

C_19_H_17_NO_2_
*M* *_r_* = 291.34
Monoclinic, 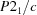

*a* = 18.9837 (10) Å
*b* = 4.740 (2) Å
*c* = 16.105 (8) Å
β = 92.927 (9)°
*V* = 1447.3 (9) Å^3^
*Z* = 4
Mo *K*α radiation
μ = 0.09 mm^−1^
*T* = 100 K
0.21 × 0.10 × 0.03 mm

#### Data collection

Rigaku AFC12 (Right) diffractometer
Absorption correction: multi-scan (*CrystalClear-SM Expert*; Rigaku, 2012 ▶) *T*_min_ = 0.982, *T*_max_ = 0.997
7947 measured reflections
3169 independent reflections
2836 reflections with *I* > 2σ(*I*)
*R*_int_ = 0.021

#### Refinement

*R*[*F* ^2^ > 2σ(*F* ^2^)] = 0.065
*wR*(*F* ^2^) = 0.175
*S* = 1.06
3169 reflections
200 parameters
6 restraints
H-atom parameters constrained
Δρ_max_ = 0.42 e Å^−3^
Δρ_min_ = −0.40 e Å^−3^

### 

Data collection: *CrystalClear-SM Expert* (Rigaku, 2012 ▶); cell refinement: *CrystalClear-SM Expert*; data reduction: *CrystalClear-SM Expert*; program(s) used to solve structure: *SHELXS97* (Sheldrick, 2008 ▶); program(s) used to refine structure: *SHELXL97* (Sheldrick, 2008 ▶); molecular graphics: *ORTEP-3 for Windows* (Farrugia, 2012 ▶); software used to prepare material for publication: *WinGX* (Farrugia, 2012 ▶), *PARST* (Nardelli, 1995 ▶) and *PLATON* (Spek, 2009 ▶).

## Supplementary Material

Crystal structure: contains datablock(s) global, I. DOI: 10.1107/S1600536812050635/su2542sup1.cif

Structure factors: contains datablock(s) I. DOI: 10.1107/S1600536812050635/su2542Isup2.hkl

Supplementary material file. DOI: 10.1107/S1600536812050635/su2542Isup3.cml

Additional supplementary materials:  crystallographic information; 3D view; checkCIF report

## Figures and Tables

**Table 1:** Hydrogen-bond geometry (Å, °)

*D*—H⋯*A*	*D*—H	H⋯*A*	*D*⋯*A*	*D*—H⋯*A*
N1—H1⋯O1	0.88	1.86	2.567 (2)	136
O2—H2⋯O1^i^	0.84	2.08	2.710 (2)	132
C11—H11⋯O2^ii^	0.95	2.55	3.327 (3)	140

Symmetry codes: (i) 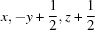; (ii) 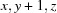.

**Fig. 1.**
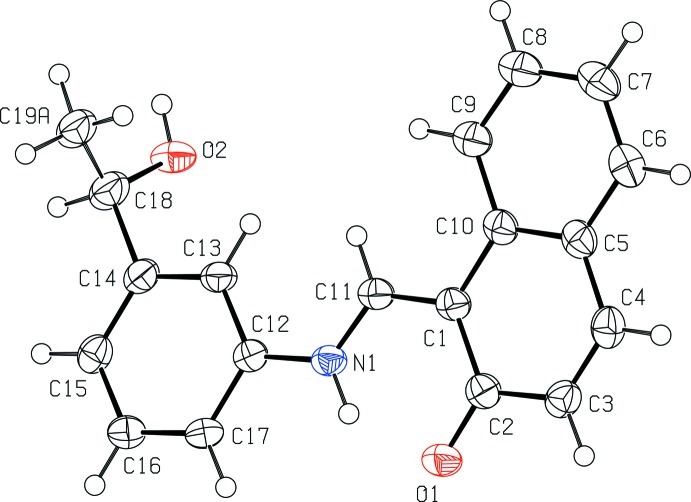
The molecular structure of the title molecule, with atom numbering. The displacement ellipsoids are drawn at the 50% probability level. Only the major component of the disordered methyl group, C19, is shown.

**Fig. 2.**
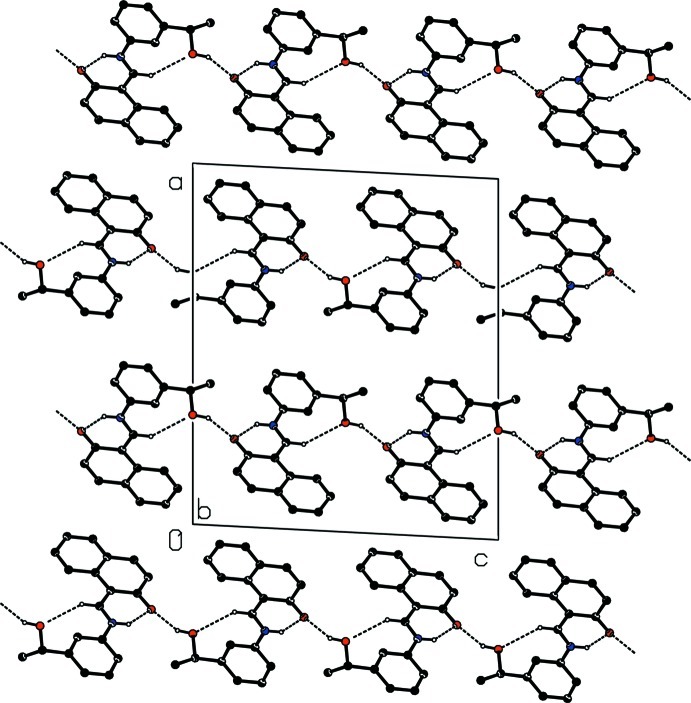
A view along the b axis of the crystal packing of the title compound. The hydrogen bonds are shown as dashed lines [H atoms not involved in the hydrogen bonding have been omitted for clarity; only the major component of the disordered methyl group, C19, is shown].

## supplementary crystallographic information

### Comment

Schiff-base complexes are considered to be among the most important stereochemical models in main group and transition metal coordination chemistry due to their preparative accessibility and structural variety (Keypour *et al.*, 2009). With the increasing incidence of deep mycosis, there has been intense emphasis on the screening of new and more effective antimicrobial drugs with low toxicity. A considerable number of Schiff-base complexes have potential biological interest, being used as more or less successful models of biological compounds (Suslick & Reinert, 1988). Not only have they played a seminal role in the development of modern coordination chemistry (Singh & Adhikari, 2012), but they can also be found at key points in the development of inorganic biochemistry, catalysis and optical materials (Tisato *et al.*, 1994). Further to our on going study on synthesis of versatile bioactive molecules we herein report the synthesis and crystal structure of the title compound.

In the title molecule, Fig.1, the C12–C17 benzene ring and the C1–C10 naphthalene ring system make a dihedral angle of 9.72 (5) °. The torsion angle of the C12—N1—C11—C1 bridging group between these rings is 179.24 (17)°. The bond lengths and angles are within the normal range (Allen *et al.*, 1987). The molecular conformation of is stabilized by an intramolecular N—H···O hydrogen bond generating an S(6) ring motif (Table 1; Bernstein *et al.*, 1995).

In the crystal, molecules are linked by C—H···O and O—H···O hydrogen bonds (Table 1 and Fig. 2), forming zigzag chains running parallel to the *ac* plane along the *c* axis direction. These chains are linked via C-H···O interactions forming a two-dimensional slab-like network lying parallel to the bc plane.

### Experimental

A mixture of 1 mmol (172 mg) 2-hydroxynaphthalene-1-carbaldehyde and 1 mmol (122 mg) 1-phenylethanol in 50 ml ethanol was refluxed for 5 h at 350 K. The reaction mixture was left to cool down at ambient temperature for 24 h when a solid precipitate was deposited. The reddish crude product was crystallized from ethanol to afford a good yield (195 mg; 67%) of high quality orange plate-like crystals suitable for X-ray diffraction analysis.

### Refinement

All the H-atoms were placed in calculated positions and treated as riding atoms: O—H = 0.84 Å, N—H = 0.88 Å, C—H = 0.95(aromatic), 0.98(methyl) and 1.00(methine) Å, with *U*_iso_(H) = k × *U*_eq_(C,*N*,*O*), where k = 1.5 for OH and methyl H atoms, and = 1.2 for other H atoms. The methyl group of the 1-phenylethanol moiety, C19, is disordered over two positions with a refined occupancy ratio of 0.775 (5):0.225 (5).

### Crystal data

**Table:**

C_19_H_17_NO_2_	*F*(000) = 616
*M**_r_* = 291.34	*D*_x_ = 1.337 Mg m^−^^3^
Monoclinic, *P*2_1_/*c*	Mo *K*α radiation, λ = 0.71075 Å
Hall symbol: -P 2ybc	Cell parameters from 3255 reflections
*a* = 18.9837 (10) Å	θ = 2.5–27.5°
*b* = 4.740 (2) Å	µ = 0.09 mm^−^^1^
*c* = 16.105 (8) Å	*T* = 100 K
β = 92.927 (9)°	Plate, orange
*V* = 1447.3 (9) Å^3^	0.21 × 0.10 × 0.03 mm
*Z* = 4	

### Data collection

**Table:**

Rigaku AFC12 (Right) diffractometer	3169 independent reflections
Radiation source: Rotating Anode	2836 reflections with *I* > 2σ(*I*)
Detector resolution: 28.5714 pixels mm^-1^	*R*_int_ = 0.021
profile data from ω–scans	θ_max_ = 27.5°, θ_min_ = 3.2°
Absorption correction: multi-scan (*CrystalClear-SM Expert*; Rigaku, 2012)	*h* = −22→24
*T*_min_ = 0.982, *T*_max_ = 0.997	*k* = −5→6
7947 measured reflections	*l* = −19→20

### Refinement

**Table:**

Refinement on *F*^2^	Primary atom site location: structure-invariant direct methods
Least-squares matrix: full	Secondary atom site location: difference Fourier map
*R*[*F*^2^ > 2σ(*F*^2^)] = 0.065	Hydrogen site location: inferred from neighbouring sites
*wR*(*F*^2^) = 0.175	H-atom parameters constrained
*S* = 1.06	*w* = 1/[σ^2^(*F*_o_^2^) + (0.0842*P*)^2^ + 0.8621*P*] where *P* = (*F*_o_^2^ + 2*F*_c_^2^)/3
3169 reflections	(Δ/σ)_max_ < 0.001
200 parameters	Δρ_max_ = 0.42 e Å^−^^3^
6 restraints	Δρ_min_ = −0.40 e Å^−^^3^

### Special details

**Table:**

Geometry. Bond distances, angles *etc*. have been calculated using the rounded fractional coordinates. All su's are estimated from the variances of the (full) variance-covariance matrix. The cell e.s.d.'s are taken into account in the estimation of distances, angles and torsion angles
Refinement. Refinement on *F*^2^ for ALL reflections except those flagged by the user for potential systematic errors. Weighted *R*-factors *wR* and all goodnesses of fit *S* are based on *F*^2^, conventional *R*-factors *R* are based on *F*, with *F* set to zero for negative *F*^2^. The observed criterion of *F*^2^ > σ(*F*^2^) is used only for calculating -*R*-factor-obs *etc*. and is not relevant to the choice of reflections for refinement. *R*-factors based on *F*^2^ are statistically about twice as large as those based on *F*, and *R*-factors based on ALL data will be even larger.

### Fractional atomic coordinates and isotropic or equivalent isotropic displacement parameters (Å^2^)

**Table:**

	*x*	*y*	*z*	*U*_iso_*/*U*_eq_	Occ. (<1)
O1	0.24195 (7)	0.6722 (3)	0.13570 (9)	0.0357 (4)	
O2	0.30336 (7)	−0.3769 (3)	0.49991 (9)	0.0353 (4)	
N1	0.28516 (8)	0.3859 (3)	0.26369 (10)	0.0273 (4)	
C1	0.19536 (9)	0.7409 (4)	0.26860 (11)	0.0274 (5)	
C2	0.19913 (9)	0.8010 (4)	0.18140 (12)	0.0300 (6)	
C3	0.15259 (10)	1.0149 (5)	0.14572 (13)	0.0334 (6)	
C4	0.10697 (10)	1.1537 (5)	0.19253 (13)	0.0349 (6)	
C5	0.10260 (9)	1.1025 (4)	0.28010 (13)	0.0319 (6)	
C6	0.05493 (10)	1.2531 (5)	0.32734 (15)	0.0384 (7)	
C7	0.05156 (11)	1.2097 (5)	0.41139 (15)	0.0409 (7)	
C8	0.09706 (11)	1.0136 (5)	0.45051 (14)	0.0382 (6)	
C9	0.14383 (10)	0.8623 (4)	0.40615 (13)	0.0337 (6)	
C10	0.14765 (9)	0.8979 (4)	0.31905 (12)	0.0286 (5)	
C11	0.23989 (9)	0.5345 (4)	0.30527 (11)	0.0272 (5)	
C12	0.33197 (9)	0.1781 (4)	0.29629 (11)	0.0259 (5)	
C13	0.32893 (10)	0.0716 (4)	0.37676 (11)	0.0301 (6)	
C14	0.37591 (10)	−0.1355 (5)	0.40543 (12)	0.0318 (6)	
C15	0.42692 (10)	−0.2333 (4)	0.35367 (12)	0.0316 (6)	
C16	0.42992 (10)	−0.1285 (4)	0.27363 (12)	0.0306 (6)	
C17	0.38249 (10)	0.0757 (4)	0.24463 (12)	0.0288 (5)	
C18	0.37040 (11)	−0.2558 (6)	0.49218 (13)	0.0427 (6)	
C19A	0.39187 (15)	−0.0604 (7)	0.55719 (17)	0.0427 (6)	0.775 (5)
C19B	0.4279 (3)	−0.288 (2)	0.5443 (5)	0.049 (3)*	0.225 (5)
H1	0.28620	0.42020	0.21010	0.0330*	
H2	0.29650	−0.40430	0.55040	0.0530*	
H4	0.07660	1.29040	0.16670	0.0420*	
H6	0.02440	1.38770	0.30060	0.0460*	
H7	0.01880	1.31150	0.44250	0.0490*	
H8	0.09560	0.98470	0.50880	0.0460*	
H9	0.17430	0.73090	0.43430	0.0400*	
H11	0.23730	0.49980	0.36310	0.0330*	
H13	0.29440	0.14120	0.41230	0.0360*	
H15	0.45980	−0.37230	0.37320	0.0380*	
H16	0.46470	−0.19700	0.23840	0.0370*	
H17	0.38460	0.14550	0.18950	0.0350*	
H18A	0.40480	−0.41550	0.49660	0.0510*	0.775 (5)
H19A	0.38530	−0.14750	0.61150	0.0640*	0.775 (5)
H19B	0.36320	0.11110	0.55180	0.0640*	0.775 (5)
H19C	0.44170	−0.01220	0.55260	0.0640*	0.775 (5)
H3	0.15420	1.05860	0.08830	0.0400*	
H18B	0.35580	−0.07110	0.51590	0.0510*	0.225 (5)
H19D	0.45850	−0.12250	0.54060	0.0730*	0.225 (5)
H19E	0.45380	−0.45720	0.52860	0.0730*	0.225 (5)
H19F	0.41320	−0.30840	0.60140	0.0730*	0.225 (5)

### Atomic displacement parameters (Å^2^)

**Table:**

	*U*^11^	*U*^22^	*U*^33^	*U*^12^	*U*^13^	*U*^23^
O1	0.0371 (7)	0.0427 (8)	0.0281 (7)	0.0014 (6)	0.0089 (6)	0.0039 (7)
O2	0.0445 (8)	0.0360 (8)	0.0263 (7)	−0.0094 (6)	0.0095 (6)	−0.0020 (6)
N1	0.0282 (7)	0.0306 (8)	0.0233 (8)	−0.0037 (6)	0.0043 (6)	0.0008 (7)
C1	0.0262 (8)	0.0290 (9)	0.0272 (9)	−0.0057 (7)	0.0032 (7)	−0.0007 (8)
C2	0.0271 (9)	0.0324 (10)	0.0307 (10)	−0.0053 (7)	0.0026 (7)	0.0008 (8)
C3	0.0320 (9)	0.0375 (11)	0.0306 (10)	−0.0048 (8)	−0.0005 (7)	0.0053 (9)
C4	0.0283 (9)	0.0351 (11)	0.0409 (12)	−0.0024 (8)	−0.0023 (8)	0.0042 (9)
C5	0.0257 (9)	0.0322 (10)	0.0380 (11)	−0.0052 (7)	0.0025 (7)	−0.0023 (9)
C6	0.0299 (10)	0.0368 (11)	0.0485 (13)	0.0009 (8)	0.0029 (8)	−0.0019 (10)
C7	0.0361 (11)	0.0398 (12)	0.0476 (13)	−0.0011 (9)	0.0112 (9)	−0.0113 (11)
C8	0.0397 (11)	0.0393 (11)	0.0364 (11)	−0.0050 (9)	0.0096 (8)	−0.0065 (10)
C9	0.0342 (10)	0.0349 (11)	0.0325 (10)	−0.0017 (8)	0.0053 (8)	−0.0030 (9)
C10	0.0244 (8)	0.0293 (9)	0.0324 (10)	−0.0066 (7)	0.0033 (7)	−0.0019 (8)
C11	0.0278 (9)	0.0290 (9)	0.0251 (9)	−0.0065 (7)	0.0039 (7)	−0.0031 (8)
C12	0.0269 (8)	0.0263 (9)	0.0245 (9)	−0.0057 (7)	0.0013 (6)	0.0001 (8)
C13	0.0283 (9)	0.0393 (11)	0.0229 (9)	−0.0063 (7)	0.0039 (7)	−0.0023 (8)
C14	0.0292 (9)	0.0411 (11)	0.0248 (9)	−0.0091 (8)	−0.0017 (7)	0.0050 (9)
C15	0.0315 (9)	0.0310 (10)	0.0316 (10)	−0.0045 (7)	−0.0037 (7)	0.0035 (9)
C16	0.0348 (10)	0.0297 (10)	0.0275 (10)	0.0001 (7)	0.0035 (7)	−0.0030 (8)
C17	0.0354 (9)	0.0295 (10)	0.0219 (9)	−0.0020 (7)	0.0047 (7)	0.0018 (8)
C18	0.0393 (9)	0.0592 (12)	0.0294 (8)	−0.0086 (8)	−0.0012 (6)	0.0102 (8)
C19A	0.0393 (9)	0.0592 (12)	0.0294 (8)	−0.0086 (8)	−0.0012 (6)	0.0102 (8)

### Geometric parameters (Å, º)

**Table:**

O1—C2	1.279 (2)	C15—C16	1.385 (3)
O2—C18	1.407 (3)	C16—C17	1.387 (3)
O2—H2	0.8400	C18—C19B	1.351 (7)
N1—C11	1.320 (2)	C18—C19A	1.441 (4)
N1—C12	1.410 (2)	C3—H3	0.9500
N1—H1	0.8800	C4—H4	0.9500
C1—C10	1.452 (3)	C6—H6	0.9500
C1—C11	1.403 (3)	C7—H7	0.9500
C1—C2	1.438 (3)	C8—H8	0.9500
C2—C3	1.445 (3)	C9—H9	0.9500
C3—C4	1.348 (3)	C11—H11	0.9500
C4—C5	1.438 (3)	C13—H13	0.9500
C5—C10	1.418 (3)	C15—H15	0.9500
C5—C6	1.406 (3)	C16—H16	0.9500
C6—C7	1.374 (3)	C17—H17	0.9500
C7—C8	1.397 (3)	C18—H18A	1.0000
C8—C9	1.370 (3)	C18—H18B	1.0000
C9—C10	1.418 (3)	C19A—H19A	0.9800
C12—C17	1.389 (3)	C19A—H19B	0.9800
C12—C13	1.395 (3)	C19A—H19C	0.9800
C13—C14	1.389 (3)	C19B—H19D	0.9800
C14—C18	1.518 (3)	C19B—H19E	0.9800
C14—C15	1.389 (3)	C19B—H19F	0.9800
			
C18—O2—H2	110.00	C4—C3—H3	119.00
C11—N1—C12	126.75 (16)	C3—C4—H4	119.00
C11—N1—H1	117.00	C5—C4—H4	119.00
C12—N1—H1	117.00	C5—C6—H6	119.00
C2—C1—C10	120.55 (16)	C7—C6—H6	119.00
C2—C1—C11	119.34 (16)	C6—C7—H7	121.00
C10—C1—C11	120.08 (16)	C8—C7—H7	120.00
C1—C2—C3	117.87 (17)	C7—C8—H8	120.00
O1—C2—C3	119.99 (18)	C9—C8—H8	119.00
O1—C2—C1	122.14 (17)	C8—C9—H9	119.00
C2—C3—C4	121.15 (19)	C10—C9—H9	119.00
C3—C4—C5	122.5 (2)	N1—C11—H11	118.00
C4—C5—C6	121.06 (18)	C1—C11—H11	118.00
C4—C5—C10	119.06 (17)	C12—C13—H13	120.00
C6—C5—C10	119.87 (19)	C14—C13—H13	120.00
C5—C6—C7	121.4 (2)	C14—C15—H15	120.00
C6—C7—C8	119.0 (2)	C16—C15—H15	120.00
C7—C8—C9	121.0 (2)	C15—C16—H16	120.00
C8—C9—C10	121.37 (18)	C17—C16—H16	120.00
C1—C10—C5	118.83 (17)	C12—C17—H17	120.00
C1—C10—C9	123.86 (17)	C16—C17—H17	120.00
C5—C10—C9	117.31 (17)	O2—C18—H18A	106.00
N1—C11—C1	123.53 (16)	O2—C18—H18B	93.00
C13—C12—C17	119.52 (17)	C14—C18—H18A	106.00
N1—C12—C13	122.93 (16)	C14—C18—H18B	93.00
N1—C12—C17	117.54 (16)	C19A—C18—H18A	106.00
C12—C13—C14	120.55 (17)	C19B—C18—H18B	95.00
C13—C14—C15	119.45 (18)	C18—C19A—H19A	109.00
C13—C14—C18	119.88 (18)	C18—C19A—H19B	109.00
C15—C14—C18	120.66 (19)	C18—C19A—H19C	109.00
C14—C15—C16	120.11 (18)	H19A—C19A—H19B	109.00
C15—C16—C17	120.46 (18)	H19A—C19A—H19C	109.00
C12—C17—C16	119.90 (18)	H19B—C19A—H19C	110.00
O2—C18—C19A	114.92 (19)	C18—C19B—H19D	110.00
C14—C18—C19B	121.5 (4)	C18—C19B—H19E	109.00
O2—C18—C19B	127.2 (4)	C18—C19B—H19F	110.00
C14—C18—C19A	113.4 (2)	H19D—C19B—H19E	109.00
O2—C18—C14	109.87 (16)	H19D—C19B—H19F	110.00
C2—C3—H3	119.00	H19E—C19B—H19F	109.00
			
C11—N1—C12—C17	−170.74 (18)	C6—C5—C10—C1	178.70 (18)
C12—N1—C11—C1	179.24 (17)	C6—C5—C10—C9	−2.2 (3)
C11—N1—C12—C13	9.9 (3)	C5—C6—C7—C8	0.6 (3)
C10—C1—C2—O1	178.02 (17)	C6—C7—C8—C9	−1.0 (3)
C10—C1—C2—C3	−1.9 (3)	C7—C8—C9—C10	−0.3 (3)
C11—C1—C2—O1	0.0 (3)	C8—C9—C10—C1	−179.09 (19)
C11—C1—C2—C3	−179.93 (17)	C8—C9—C10—C5	1.9 (3)
C11—C1—C10—C9	1.8 (3)	N1—C12—C13—C14	179.28 (18)
C2—C1—C11—N1	−1.1 (3)	C17—C12—C13—C14	0.0 (3)
C10—C1—C11—N1	−179.17 (17)	N1—C12—C17—C16	179.89 (17)
C11—C1—C10—C5	−179.16 (17)	C13—C12—C17—C16	−0.8 (3)
C2—C1—C10—C5	2.8 (3)	C12—C13—C14—C15	1.0 (3)
C2—C1—C10—C9	−176.24 (17)	C12—C13—C14—C18	−178.05 (19)
O1—C2—C3—C4	180.0 (2)	C13—C14—C15—C16	−1.2 (3)
C1—C2—C3—C4	−0.1 (3)	C18—C14—C15—C16	177.85 (19)
C2—C3—C4—C5	1.2 (3)	C13—C14—C18—O2	59.0 (3)
C3—C4—C5—C6	179.3 (2)	C13—C14—C18—C19A	−71.1 (3)
C3—C4—C5—C10	−0.2 (3)	C15—C14—C18—O2	−120.0 (2)
C4—C5—C6—C7	−178.5 (2)	C15—C14—C18—C19A	109.8 (3)
C10—C5—C6—C7	1.0 (3)	C14—C15—C16—C17	0.4 (3)
C4—C5—C10—C1	−1.8 (3)	C15—C16—C17—C12	0.6 (3)
C4—C5—C10—C9	177.36 (18)		

### Hydrogen-bond geometry (Å, º)

**Table:**

*D*—H···*A*	*D*—H	H···*A*	*D*···*A*	*D*—H···*A*
N1—H1···O1	0.88	1.86	2.567 (2)	136
O2—H2···O1^i^	0.84	2.08	2.710 (2)	132
C11—H11···O2^ii^	0.95	2.55	3.327 (3)	140

Symmetry codes: (i) *x*, −*y*+1/2, *z*+1/2; (ii) *x*, *y*+1, *z*.
